# Invasive Mycobacterium bovis Infection Outside the Genitourinary Tract Following Bacille Calmette-Guerin Therapy for Non-muscle Invasive Bladder Cancer

**DOI:** 10.7759/cureus.63613

**Published:** 2024-07-01

**Authors:** Debduti Mukhopadhyay, Samuel Booth, Taher Sbitli, Kevin T Shiley, Diana Pomakova

**Affiliations:** 1 Internal Medicine, University at Buffalo Jacobs School of Medicine and Biomedical Sciences, Buffalo, USA; 2 Infectious Disease, Mercy Hospital of Buffalo, Buffalo, USA

**Keywords:** tuberculosis, mycobaterium avium complex, bcg, non-muscle invasive bladder cancer, bladder cancer

## Abstract

Bladder cancer significantly impacts global health, particularly non-muscle-invasive bladder cancer (NMIBC), which is typically treated with transurethral resection of bladder tumor (TURBT) and intravesical Bacillus Calmette-Guérin (BCG) therapy. While there is evidence that BCG can effectively prevent tumor recurrence and progression, it can cause adverse effects, including disseminated infection, necessitating the exclusion of active tuberculosis and the assessment of immunosuppressive conditions before treatment. We present two cases of disseminated BCG infection. The first involves an 85-year-old male who developed an abscess in his right thigh post-BCG therapy, successfully treated with isoniazid (INH), ethambutol, and rifampin. The second case is a 63-year-old male who, three years post-BCG therapy and abdominal aortic aneurysm repair, developed a right psoas abscess and a mycotic aneurysm. He was also treated with ethambutol, INH, and rifampin, in addition to surgical intervention. Effective management of BCG-related infections requires early identification of *Mycobacterium bovis*, a multidisciplinary approach, thorough pre-treatment evaluations, and aggressive treatment strategies, including anti-tubercular drugs and surgical intervention as necessary.

## Introduction

Bladder cancer ranks among the top ten most prevalent cancers worldwide, posing significant challenges to healthcare systems due to its impact on morbidity and mortality. Its occurrence is influenced by demographic shifts such as population growth and aging, along with exposure to key risk factors, notably tobacco smoking [1]. The recommended approach for treating non-muscle-invasive bladder cancer (NMIBC), begins with complete tumor removal through transurethral resection of bladder tumor (TURBT) followed by intravesical Bacillus Calmette-Guérin (BCG). BCG effectively prevents tumor recurrence or progression, as evidenced by the first controlled trial conducted in 1980, and a 2023 metanalysis showing a significant reduction in odds of progression [2]. BCG therapy, introduced by Alvaro Morales in 1972, involves tailored induction and maintenance phases based on tumor risk levels, with specific administration protocols. Predictors of treatment failure include the neutrophil-to-lymphocyte (NtL) ratio and the tumor microenvironment, which can impact immune response and treatment outcomes. Despite its efficacy, BCG therapy may cause adverse effects, both locally and systemically [3,4]. Disseminated BCG infection also known as “BCGitis,” may lead to complications in the lungs, liver, bones, and joints necessitating the exclusion of active tuberculosis and the assessment of systemic risk factors for immunosuppression before initiating therapy. Instillation is contraindicated in vesical mucosa breaches, recent TURBT, symptomatic bacterial cystitis, gross hematuria, or traumatic urological procedures. Several strategies, such as adjusting the mycobacterial load, short-term anti-TB drug therapy, or symptomatic management of cystitis, have been explored to prevent infectious complications with limited success [5]. These cases were presented at the New York Chapter of the American College of Physicians (NYACP) in Rochester, United States, on October 2023.

## Case presentation

Case 1

**Figure 1:**
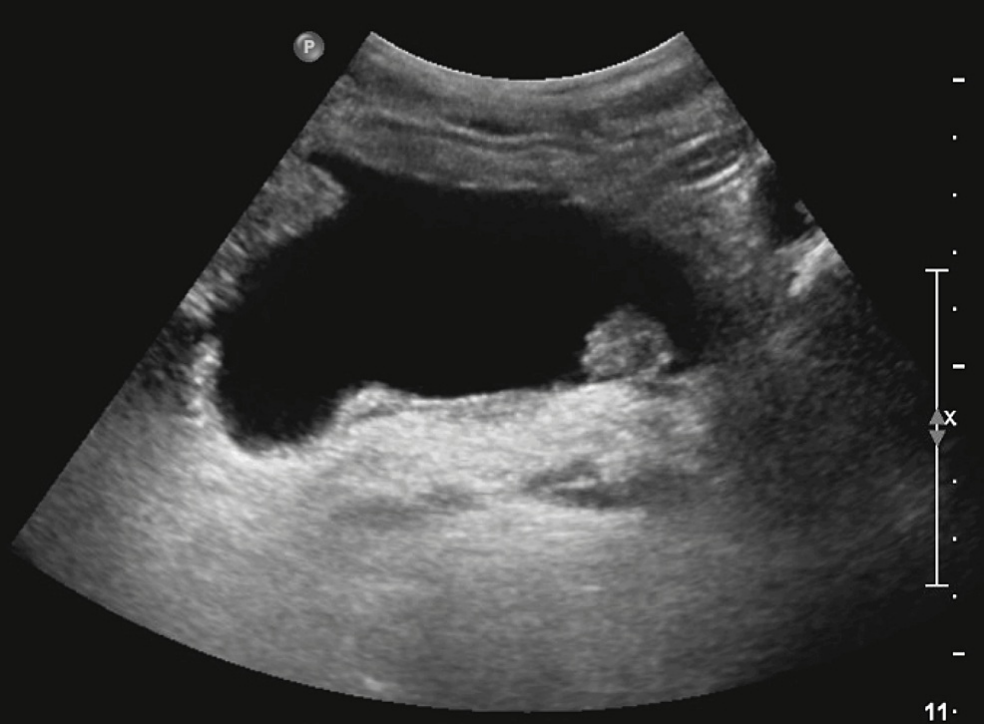
Ultrasound of the bladder showing a lobulated polypoid lesion

**Figure 2:**
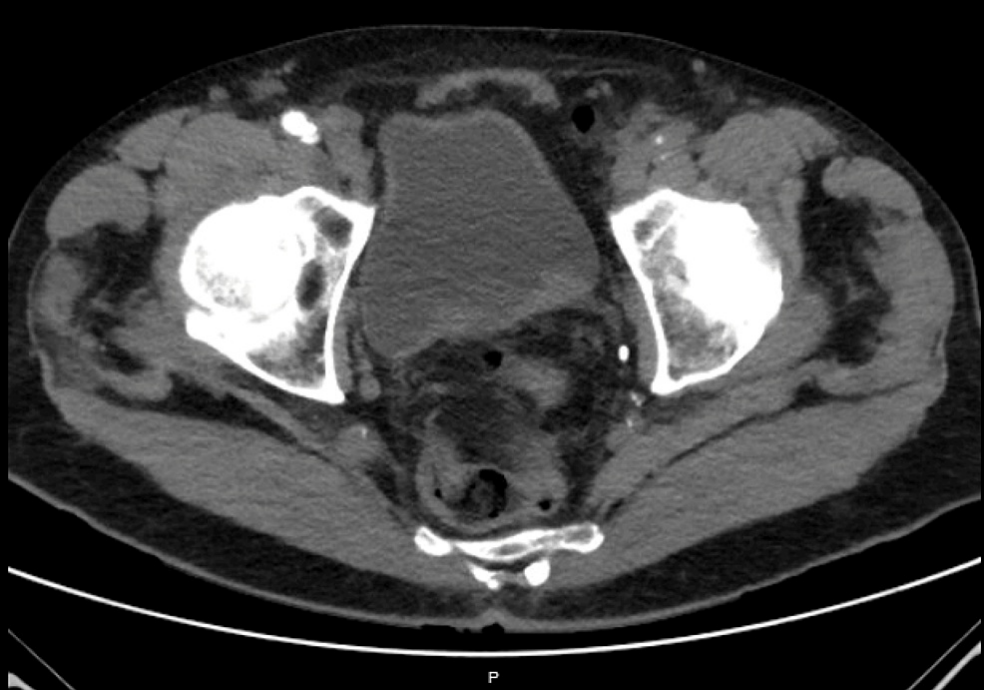
CT urogram showing bladder lobulated polypoid lesion

An 85-year-old Caucasian male, a former smoker, presented with progressively worsening dyspnea on exertion. Laboratory tests revealed a hemoglobin of 5.9 g/dl and an iron saturation of <10%. Cardiopulmonary causes of dyspnea and gastrointestinal causes of active bleeding were ruled out. He received treatment with packed red blood cells and intravenous iron, with improvement. During a follow-up visit, he reported increased urinary frequency and urgency, poor urinary stream, nocturia, and intermittent hematuria. Imaging confirmed the presence of a lobulated polypoid lesion in the bladder (Figures 1, 2). Subsequently, he underwent a TURBT, which revealed multifocal tumors on the posterior wall of the bladder. Histology revealed non-invasive high-grade Ta papillary urothelial carcinoma with no muscularis propria identified. The patient was initially offered BCG therapy, but he declined it. A staging TURBT done two months later revealed tumor recurrence of low-grade papillary urothelial carcinoma. A follow-up surveillance cystoscopy done three months later revealed multifocal tumor recurrence, which prompted a third TURBT and the initiation of a six-week induction course of intravesical BCG therapy, followed by maintenance. He developed superficial patchy cystitis consistent with BCG irritation, and therapy was temporarily stopped. Two months after this, he developed a complex multiloculated collection in the right medial thigh, extending from the superficial compartment into the deep muscular compartments, requiring drainage. The collection was aspirated, and a diagnosis of pyogenic abscess was made. Final cultures grew *Mycobacterium bovis* (*M. Bovis*). He was treated with a combination of isoniazid (INH) - 300 mg PO daily, ethambutol - 1200 mg PO daily, and rifampin - 300 mg PO twice daily for two months followed by rifampin and INH for seven more months. At the juncture of this report, he has been off antibiotics for one month and is doing well.

Case 2

**Figure 3:**
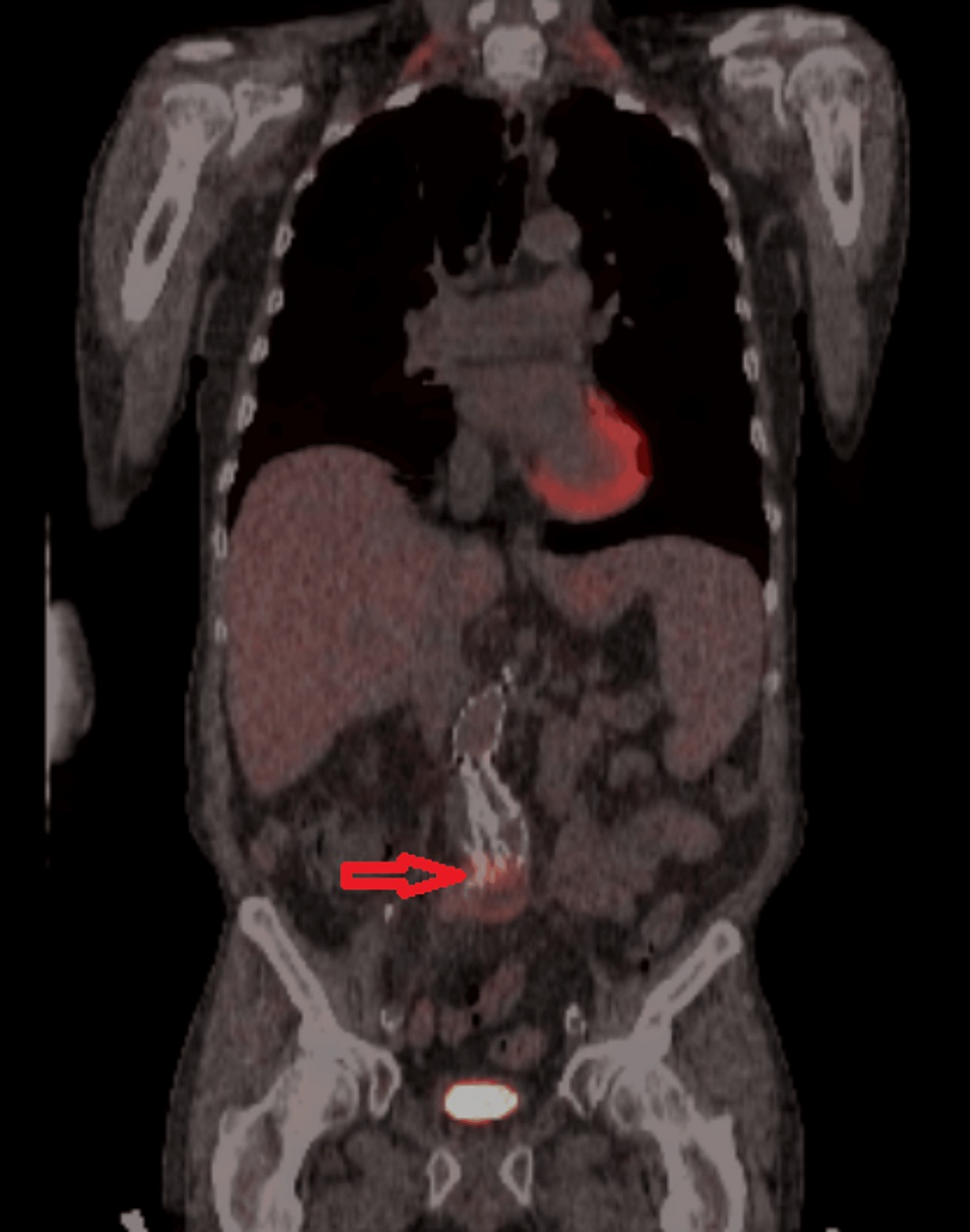
Heterogenous fluorodeoxyglucose (FDG) uptake involving the enlarging abdominal aortic endoleak (red arrow) at the site of the previous stenting

A 63-year-old Caucasian male, a former smoker, treated with intravesical BCG for bladder cancer five weeks after an abdominal aortic aneurysm repair with grafting, presented three years later with weight loss, low-grade fevers, lumbar and flank pain. Imaging showed a right psoas fluid collection, and an enlarging abdominal aortic endoleak (Figure 3) at the site of previous stenting requiring drainage and washout. Despite starting empiric broad-spectrum antibiotics, his fevers and rigors persisted. Fluid cultures showed the presence of acid-fast bacilli (AFB). A PCR study confirmed the pathogen to be *M. bovis*. The pathology of the peri-aortic abscess showed caseating granulomatous inflammation. The patient began treatment with ethambutol, INH, and rifampin, deferring graft removal due to malnutrition. His symptoms and weight improved. After six months, he developed a mycotic aneurysm in the lower abdominal aorta and right iliac artery, necessitating graft excision and a right axillo-femoral bypass. Ethambutol was discontinued after three months, with rifampin and isoniazid continuing for 12 months. No recurrence of infection was noted one-year post-treatment.

## Discussion

Despite being utilized in treating NMIBC for nearly half a century, the precise mechanism of action of BCG remains unclear. It is hypothesized that BCG targets the malignant cells by attaching to fibronectin leading to internalization. The normal urothelial tissue has negatively charged proteins that repel the similarly charged BCG [6]. Genitourinary symptoms, low-grade fever, and malaise are common following instillation and typically resolve within a few days; a history of systemic BCG infection is a contraindication to restarting immunotherapy [7].

The symptoms of cystitis in our first patient remain consistent with reported local symptoms of intravesical BCG. For our second patient, the associated timeline following therapy highlights the occurrence of late systemic complications. Histological examination revealing granulomas can be pivotal in establishing a diagnosis. The treatment team must remain vigilant for BCG-related mycotic aneurysms and prosthetic joint infections to aid in prompt management leading to a favorable outcome as noticed in our patient. Mortality rates vary depending on the affected organ system, with vascular complications carrying the highest mortality risk [8,9].

A mycotic aneurysm is a serious complication of BCG instillation with the first documented case in 1988. The treatment for that case involved excision and grafting followed by antituberculous medications [10]. Mycotic aneurysms secondary to BCG instillation have a poor prognosis, regardless of whether they are managed by open repair or endovascular techniques [11]. They have been reported in patients between 58 years and 71 years with sizes ranging from 1 cm to 8 cm, typically appearing saccular and thin-walled on radiographic imaging. The most affected vessel is the aorta which aligns with the clinical presentation of our patient [12]. Psoas muscle involvement is usually seen in those with infrarenal mycotic aneurysms, with the exception of one case involving an aneurysm of the aortic arch and an isolated psoas muscle abscess [13,14].

The typical treatment regimen includes INH (300 mg), rifampin (600 mg), and ethambutol (1200 mg) for 6-12 months despite some reports of isoniazid resistance present in the literature. Pyrazinamide is excluded due to inherent resistance in all *M. bovis* strains. Ofloxacin, doxycycline, or moxifloxacin are used as alternatives in case of side effects. In instances of vascular graft infections, surgical intervention is typically necessary. Corticosteroids are used when hypersensitivity is suspected as the cause of the systemic infection [15-17]. Improvement solely with ofloxacin, ethambutol, and steroids has also been reported [18].

## Conclusions

Our cases underscore the necessity for healthcare providers to maintain a high index of suspicion for BCG-related infections, particularly in patients presenting with nonspecific symptoms post-BCG therapy. Effective management requires a multidisciplinary approach involving timely diagnosis and appropriate anti-tubercular therapy. Early identification of *M. bovis*, through fluid aspiration and molecular techniques, is crucial for initiating targeted treatment. Moreover, the prolonged nature of anti-tubercular regimens and the potential for significant side effects necessitate diligent monitoring and patient compliance. The absence of a proven prophylaxis protocol for systemic BCG infection reinforces the importance of thorough pre-treatment evaluations, including the exclusion of active tuberculosis and the assessment of immunosuppressive conditions. Our findings advocate for heightened awareness and readiness to implement aggressive treatment strategies, including the potential for surgical intervention in cases of vascular involvement.
